# Diethyl 2-{4-diethyl­amino-2-[(dimethyl­carbamothio­yl)­oxy]benzyl­idene}malonate

**DOI:** 10.1107/S1600536811024305

**Published:** 2011-06-30

**Authors:** Dan Sun, Xiao-Xing Fan, Shi-Yao Yang, Hong Zheng

**Affiliations:** a The Key Laboratory of Analytical Sciences, Ministry of Education, College of Chemistry and Chemical Engineering, Xiamen University, Xiamen 361005, People’s Republic of China; b Department of Chemistry, College of Chemistry and Chemical Engineering, Xiamen University, Xiamen 361005, People’s Republic of China

## Abstract

In the title compound, C_21_H_30_N_2_O_5_S, the plane of the dimeth­yl–thio­carbamic group makes a dihedral angle of 78.41 (7)° with the central benzene ring. One of the carbonyl groups in the α,β-unsaturated malonate side chain makes a dihedral angle of 8.73 (10)° with the central benzene ring, while the other carbonyl group makes a dihedral angle of 81.52 (8)°.

## Related literature

For related structures, see: Jiang & Wang (2009 ▶); Kim & Swager (2003 ▶). For hypochlorous acid probes, see: Sun *et al.* (2008 ▶).
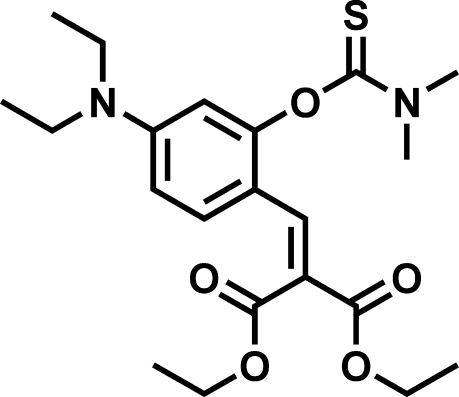


## Experimental

### 

#### Crystal data

C_21_H_30_N_2_O_5_S
*M* *_r_* = 422.53
Monoclinic, 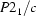

*a* = 14.2704 (6) Å
*b* = 9.2716 (4) Å
*c* = 25.9206 (8) Å
β = 139.588 (1)°
*V* = 2223.30 (15) Å^3^
*Z* = 4
Mo *K*α radiation
μ = 0.18 mm^−1^
*T* = 173 K
0.40 × 0.37 × 0.07 mm

#### Data collection

Bruker APEX area-detector diffractometer
Absorption correction: multi-scan (*SADABS*; Bruker, 2002 ▶) *T*_min_ = 0.932, *T*_max_ = 0.988
25540 measured reflections
5414 independent reflections
4778 reflections with *I* > 2σ(*I*)
*R*_int_ = 0.037

#### Refinement

*R*[*F* ^2^ > 2σ(*F* ^2^)] = 0.068
*wR*(*F* ^2^) = 0.155
*S* = 1.18
5414 reflections
268 parameters
H-atom parameters constrained
Δρ_max_ = 0.39 e Å^−3^
Δρ_min_ = −0.29 e Å^−3^

### 

Data collection: *SMART* (Bruker, 2002 ▶); cell refinement: *SAINT* (Bruker, 2002 ▶); data reduction: *SAINT*; program(s) used to solve structure: *SHELXS97* (Sheldrick, 2008 ▶); program(s) used to refine structure: *SHELXL97* (Sheldrick, 2008 ▶); molecular graphics: *DIAMOND* (Brandenburg, 2011 ▶); software used to prepare material for publication: *SHELXL97*.

## Supplementary Material

Crystal structure: contains datablock(s) I, global. DOI: 10.1107/S1600536811024305/kj2176sup1.cif

Structure factors: contains datablock(s) I. DOI: 10.1107/S1600536811024305/kj2176Isup2.hkl

Supplementary material file. DOI: 10.1107/S1600536811024305/kj2176Isup3.cml

Additional supplementary materials:  crystallographic information; 3D view; checkCIF report

## Figures and Tables

**Fig. 1.**
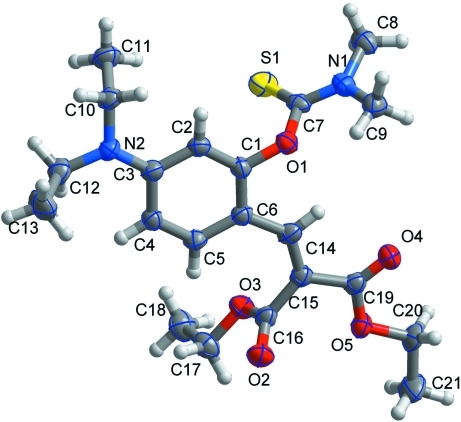
Thermal ellipsoid plot of the title compound. Displacement ellipsoids are drawn at 50% probabability level.

## supplementary crystallographic information

### Comment

Reactive oxygen species (ROS) are known to be essential to several biological functions and hypochlorous acid (HOCl) is one of the biologically important ROS. Therefore, the determination of hypochlorous acid is very important for biological research, but it is still a challenge for the design and synthesis of highly specific and sensitive probes for hypochlorous acid (Sun *et al.*, 2008). We have therefore synthesized the title compound, and investigated its fluorescent spectral response to hypochlorous acid, and further experiments show that the title compound can react with hypochlorous acid, which result in a remarkable fluorescence enhancement. Therefore, the title compound can serve as a sensitive fluorescent probe for the determination of hypochlorous acid. The chemical structures of related compounds (Jiang & Wang, 2009; Kim, & Swager 2003) have been reported, yet the crystal structures have not been reported. The title compound was obtained by two steps of chemical reactions. An X-ray crystal structure determination of the molecular structure of title compound was carried out to determine its conformation.

In the molecular structure of title compound (Fig.1), the plane of dimethyl-thiocarbamic group with the central benzene ring make a dihedral angle of 78.41 (7)° (Brandenburg, 2011). In addition, one carbonyl group in the a,b-unsaturated malonate side chain, make a dihedral angle of 8.73 (10)° with central benzene ring. The other carbonyl group, however, with the central benzene ring make a dihedral angle of 81.52 (8)°. The structure is therefore a conjugate electron system with "push-pull" substituent pairs, and shows strong intramolecular charge transfer (ICT) effects.

### Experimental

The title compound was obtained from a two step synthesis. First, the reaction of 4-(diethylamino) salicylaldehyde and dimethyl thiocarbamoyl chloride under potassium carbonate in a DMF solution gave dimethyl-thiocarbamic acid *O*-(5-diethylamino-2-formyl-phenyl) ester. This product was refluxed for 3 h with diethyl malonate under the catalysis of piperidine in acetonitrile. After removal of the solvent, the residue was purified by flash chromatography with dichloromethane / petroleum ether as eluent to afford the title compound in 35% yield. The title compound exhibits an absorption maximum at 400 nm and in addition it also displays a minimal fluorescence emission at 509 nm with maximum excitation at 403 nm. Single crystals were obtained by slow evaporation of a dichloromethane / petroleum ether (5/1, *v*/*v*).

### Refinement

The aromatic H atoms were generated geometrically (C—H 0.93, N—H 0.86 Å) and were allowed to ride on their parent atoms in the riding model approximations, with their displacement factors set to 1.2 times those of the parent atoms.

### Crystal data

**Table:**

C_21_H_30_N_2_O_5_S	*F*(000) = 904
*M**_r_* = 422.53	*D*_x_ = 1.262 Mg m^−^^3^*D*_m_ = 1.262 Mg m^−^^3^*D*_m_ measured by not measured
Monoclinic, *P*2_1_/*c*	Mo *K*α radiation, λ = 0.71073 Å
Hall symbol: -P 2ybc	Cell parameters from 6772 reflections
*a* = 14.2704 (6) Å	θ = 2.4–28.0°
*b* = 9.2716 (4) Å	µ = 0.18 mm^−^^1^
*c* = 25.9206 (8) Å	*T* = 173 K
β = 139.588 (1)°	Plate, yellow
*V* = 2223.30 (15) Å^3^	0.40 × 0.37 × 0.07 mm
*Z* = 4	

### Data collection

**Table:**

Bruker APEX area-detector diffractometer	5414 independent reflections
Radiation source: fine-focus sealed tube	4778 reflections with *I* > 2σ(*I*)
graphite	*R*_int_ = 0.037
φ and ω scans	θ_max_ = 28.8°, θ_min_ = 1.6°
Absorption correction: multi-scan (*SADABS*; Bruker, 2002)	*h* = −19→19
*T*_min_ = 0.932, *T*_max_ = 0.988	*k* = −12→11
25540 measured reflections	*l* = −34→33

### Refinement

**Table:**

Refinement on *F*^2^	Primary atom site location: structure-invariant direct methods
Least-squares matrix: full	Secondary atom site location: difference Fourier map
*R*[*F*^2^ > 2σ(*F*^2^)] = 0.068	Hydrogen site location: inferred from neighbouring sites
*wR*(*F*^2^) = 0.155	H-atom parameters constrained
*S* = 1.18	*w* = 1/[σ^2^(*F*_o_^2^) + (0.0604*P*)^2^ + 1.2811*P*] where *P* = (*F*_o_^2^ + 2*F*_c_^2^)/3
5414 reflections	(Δ/σ)_max_ < 0.001
268 parameters	Δρ_max_ = 0.39 e Å^−^^3^
0 restraints	Δρ_min_ = −0.29 e Å^−^^3^

### Special details

**Table:**

Geometry. All e.s.d.'s (except the e.s.d. in the dihedral angle between two l.s. planes) are estimated using the full covariance matrix. The cell e.s.d.'s are taken into account individually in the estimation of e.s.d.'s in distances, angles and torsion angles; correlations between e.s.d.'s in cell parameters are only used when they are defined by crystal symmetry. An approximate (isotropic) treatment of cell e.s.d.'s is used for estimating e.s.d.'s involving l.s. planes.
Refinement. Refinement of *F*^2^ against ALL reflections. The weighted *R*-factor *wR* and goodness of fit *S* are based on *F*^2^, conventional *R*-factors *R* are based on *F*, with *F* set to zero for negative *F*^2^. The threshold expression of *F*^2^ > σ(*F*^2^) is used only for calculating *R*-factors(gt) *etc*. and is not relevant to the choice of reflections for refinement. *R*-factors based on *F*^2^ are statistically about twice as large as those based on *F*, and *R*- factors based on ALL data will be even larger.

### Fractional atomic coordinates and isotropic or equivalent isotropic displacement parameters (Å^2^)

**Table:**

	*x*	*y*	*z*	*U*_iso_*/*U*_eq_	
S1	0.26921 (7)	0.44597 (7)	0.62902 (4)	0.03779 (17)	
O1	0.46394 (15)	0.63622 (16)	0.74213 (8)	0.0260 (3)	
O2	0.67228 (17)	0.44354 (18)	1.03491 (9)	0.0376 (4)	
O3	0.51195 (16)	0.28130 (16)	0.94122 (9)	0.0295 (3)	
O4	0.82337 (16)	0.34284 (18)	0.94609 (9)	0.0350 (4)	
O5	0.83126 (16)	0.24105 (17)	1.02732 (9)	0.0320 (4)	
N1	0.4991 (2)	0.5538 (2)	0.67853 (11)	0.0308 (4)	
N2	0.07452 (18)	0.8351 (2)	0.67478 (10)	0.0281 (4)	
C1	0.3890 (2)	0.6452 (2)	0.75718 (11)	0.0229 (4)	
C2	0.2706 (2)	0.7321 (2)	0.70888 (11)	0.0248 (4)	
H2A	0.2389	0.7792	0.6651	0.030*	
C3	0.1956 (2)	0.7521 (2)	0.72368 (11)	0.0241 (4)	
C4	0.2515 (2)	0.6825 (2)	0.79130 (11)	0.0248 (4)	
H4A	0.2057	0.6963	0.8045	0.030*	
C5	0.3701 (2)	0.5958 (2)	0.83781 (11)	0.0244 (4)	
H5A	0.4033	0.5492	0.8822	0.029*	
C6	0.4447 (2)	0.5725 (2)	0.82317 (11)	0.0235 (4)	
C7	0.4143 (2)	0.5470 (2)	0.68376 (12)	0.0259 (4)	
C8	0.4655 (3)	0.4683 (3)	0.61905 (15)	0.0391 (6)	
H8A	0.4317	0.3727	0.6155	0.047*	
H8B	0.5524	0.4581	0.6342	0.047*	
H8C	0.3897	0.5165	0.5673	0.047*	
C9	0.6294 (2)	0.6407 (3)	0.73122 (14)	0.0372 (5)	
H9A	0.6352	0.7007	0.7647	0.045*	
H9B	0.6268	0.7027	0.6995	0.045*	
H9C	0.7141	0.5774	0.7650	0.045*	
C10	0.0145 (2)	0.9012 (2)	0.60339 (12)	0.0304 (5)	
H10A	0.0947	0.9353	0.6149	0.037*	
H10B	−0.0435	0.9864	0.5892	0.037*	
C11	−0.0791 (3)	0.8002 (3)	0.53356 (13)	0.0416 (6)	
H11A	−0.1153	0.8500	0.4876	0.050*	
H11B	−0.1609	0.7688	0.5205	0.050*	
H11C	−0.0222	0.7160	0.5470	0.050*	
C12	−0.0149 (2)	0.8433 (3)	0.68272 (14)	0.0352 (5)	
H12A	−0.0061	0.7518	0.7058	0.042*	
H12B	−0.1171	0.8536	0.6291	0.042*	
C13	0.0234 (4)	0.9656 (3)	0.7337 (2)	0.0611 (8)	
H13A	−0.0464	0.9705	0.7328	0.073*	
H13B	0.0211	1.0562	0.7132	0.073*	
H13C	0.1206	0.9505	0.7884	0.073*	
C14	0.5714 (2)	0.4841 (2)	0.86988 (11)	0.0250 (4)	
H14A	0.6099	0.4850	0.8521	0.030*	
C15	0.6448 (2)	0.4009 (2)	0.93361 (11)	0.0248 (4)	
C16	0.6132 (2)	0.3805 (2)	0.97606 (11)	0.0259 (4)	
C17	0.4749 (3)	0.2533 (3)	0.97892 (15)	0.0389 (5)	
H17A	0.4430	0.3434	0.9826	0.047*	
H17B	0.5598	0.2153	1.0333	0.047*	
C18	0.3562 (3)	0.1456 (3)	0.92939 (18)	0.0491 (7)	
H18A	0.3239	0.1292	0.9514	0.059*	
H18B	0.3912	0.0547	0.9293	0.059*	
H18C	0.2753	0.1817	0.8748	0.059*	
C19	0.7746 (2)	0.3272 (2)	0.96794 (12)	0.0267 (4)	
C20	0.9633 (3)	0.1675 (3)	1.06771 (14)	0.0382 (5)	
H20A	1.0344	0.2375	1.0839	0.046*	
H20B	0.9428	0.0949	1.0321	0.046*	
C21	1.0204 (3)	0.0966 (4)	1.13851 (18)	0.0591 (8)	
H21A	1.1102	0.0457	1.1676	0.071*	
H21B	0.9491	0.0276	1.1217	0.071*	
H21C	1.0400	0.1696	1.1731	0.071*	

### Atomic displacement parameters (Å^2^)

**Table:**

	*U*^11^	*U*^22^	*U*^33^	*U*^12^	*U*^13^	*U*^23^
S1	0.0414 (3)	0.0372 (3)	0.0373 (3)	−0.0122 (3)	0.0306 (3)	−0.0120 (2)
O1	0.0245 (7)	0.0321 (8)	0.0240 (7)	−0.0033 (6)	0.0192 (6)	−0.0039 (6)
O2	0.0341 (8)	0.0457 (10)	0.0272 (8)	−0.0033 (7)	0.0217 (7)	−0.0105 (7)
O3	0.0340 (8)	0.0281 (8)	0.0303 (7)	−0.0015 (6)	0.0255 (7)	−0.0032 (6)
O4	0.0330 (8)	0.0432 (9)	0.0327 (8)	0.0092 (7)	0.0260 (7)	0.0039 (7)
O5	0.0297 (8)	0.0341 (8)	0.0314 (8)	0.0121 (6)	0.0230 (7)	0.0094 (6)
N1	0.0369 (10)	0.0302 (9)	0.0342 (9)	0.0037 (8)	0.0295 (9)	0.0027 (8)
N2	0.0257 (8)	0.0313 (9)	0.0232 (8)	0.0074 (7)	0.0174 (7)	0.0059 (7)
C1	0.0228 (9)	0.0241 (9)	0.0217 (9)	−0.0036 (8)	0.0169 (8)	−0.0045 (8)
C2	0.0250 (9)	0.0272 (10)	0.0197 (9)	−0.0002 (8)	0.0163 (8)	0.0015 (8)
C3	0.0224 (9)	0.0219 (9)	0.0223 (9)	−0.0002 (7)	0.0155 (8)	−0.0015 (7)
C4	0.0247 (9)	0.0279 (10)	0.0244 (9)	0.0005 (8)	0.0194 (9)	−0.0002 (8)
C5	0.0255 (9)	0.0243 (10)	0.0197 (9)	0.0003 (8)	0.0161 (8)	0.0008 (8)
C6	0.0214 (9)	0.0225 (10)	0.0211 (9)	−0.0009 (7)	0.0147 (8)	−0.0026 (7)
C7	0.0296 (10)	0.0242 (10)	0.0233 (9)	0.0044 (8)	0.0201 (9)	0.0036 (8)
C8	0.0589 (16)	0.0341 (12)	0.0474 (14)	0.0074 (11)	0.0469 (14)	0.0023 (10)
C9	0.0343 (12)	0.0456 (14)	0.0399 (12)	0.0043 (10)	0.0305 (11)	0.0056 (11)
C10	0.0292 (10)	0.0287 (10)	0.0258 (10)	0.0060 (9)	0.0189 (9)	0.0054 (8)
C11	0.0343 (12)	0.0497 (15)	0.0268 (11)	0.0004 (11)	0.0193 (10)	−0.0016 (10)
C12	0.0271 (10)	0.0404 (13)	0.0355 (11)	0.0094 (10)	0.0231 (10)	0.0106 (10)
C13	0.084 (2)	0.0569 (18)	0.075 (2)	0.0151 (17)	0.069 (2)	0.0026 (16)
C14	0.0229 (9)	0.0252 (10)	0.0229 (9)	−0.0020 (8)	0.0164 (9)	−0.0042 (8)
C15	0.0219 (9)	0.0240 (10)	0.0207 (9)	−0.0010 (8)	0.0141 (8)	−0.0050 (8)
C16	0.0220 (9)	0.0239 (10)	0.0205 (9)	0.0069 (8)	0.0130 (8)	0.0021 (8)
C17	0.0528 (15)	0.0358 (12)	0.0456 (13)	0.0028 (11)	0.0423 (13)	0.0016 (10)
C18	0.0492 (15)	0.0507 (16)	0.0628 (17)	−0.0013 (13)	0.0469 (15)	0.0011 (14)
C19	0.0253 (10)	0.0251 (10)	0.0232 (9)	−0.0005 (8)	0.0167 (9)	−0.0052 (8)
C20	0.0327 (12)	0.0415 (13)	0.0354 (12)	0.0169 (10)	0.0246 (11)	0.0096 (10)
C21	0.0573 (18)	0.071 (2)	0.0534 (17)	0.0381 (16)	0.0433 (16)	0.0348 (15)

### Geometric parameters (Å, °)

**Table:**

S1—C7	1.645 (2)	C9—H9C	0.9800
O1—C7	1.358 (2)	C10—C11	1.502 (3)
O1—C1	1.393 (2)	C10—H10A	0.9900
O2—C16	1.192 (2)	C10—H10B	0.9900
O3—C16	1.327 (3)	C11—H11A	0.9800
O3—C17	1.445 (3)	C11—H11B	0.9800
O4—C19	1.194 (3)	C11—H11C	0.9800
O5—C19	1.329 (3)	C12—C13	1.493 (4)
O5—C20	1.451 (3)	C12—H12A	0.9900
N1—C7	1.317 (3)	C12—H12B	0.9900
N1—C9	1.450 (3)	C13—H13A	0.9800
N1—C8	1.452 (3)	C13—H13B	0.9800
N2—C3	1.360 (3)	C13—H13C	0.9800
N2—C12	1.441 (3)	C14—C15	1.336 (3)
N2—C10	1.456 (3)	C14—H14A	0.9500
C1—C2	1.360 (3)	C15—C19	1.476 (3)
C1—C6	1.394 (3)	C15—C16	1.485 (3)
C2—C3	1.397 (3)	C17—C18	1.484 (4)
C2—H2A	0.9500	C17—H17A	0.9900
C3—C4	1.413 (3)	C17—H17B	0.9900
C4—C5	1.363 (3)	C18—H18A	0.9800
C4—H4A	0.9500	C18—H18B	0.9800
C5—C6	1.394 (3)	C18—H18C	0.9800
C5—H5A	0.9500	C20—C21	1.478 (3)
C6—C14	1.439 (3)	C20—H20A	0.9900
C8—H8A	0.9800	C20—H20B	0.9900
C8—H8B	0.9800	C21—H21A	0.9800
C8—H8C	0.9800	C21—H21B	0.9800
C9—H9A	0.9800	C21—H21C	0.9800
C9—H9B	0.9800		
			
C7—O1—C1	119.27 (16)	C10—C11—H11C	109.5
C16—O3—C17	115.70 (17)	H11A—C11—H11C	109.5
C19—O5—C20	115.40 (17)	H11B—C11—H11C	109.5
C7—N1—C9	123.26 (19)	N2—C12—C13	113.5 (2)
C7—N1—C8	119.8 (2)	N2—C12—H12A	108.9
C9—N1—C8	116.97 (19)	C13—C12—H12A	108.9
C3—N2—C12	122.11 (17)	N2—C12—H12B	108.9
C3—N2—C10	121.58 (17)	C13—C12—H12B	108.9
C12—N2—C10	115.53 (17)	H12A—C12—H12B	107.7
C2—C1—O1	117.30 (17)	C12—C13—H13A	109.5
C2—C1—C6	124.39 (18)	C12—C13—H13B	109.5
O1—C1—C6	118.19 (17)	H13A—C13—H13B	109.5
C1—C2—C3	120.01 (18)	C12—C13—H13C	109.5
C1—C2—H2A	120.0	H13A—C13—H13C	109.5
C3—C2—H2A	120.0	H13B—C13—H13C	109.5
N2—C3—C2	120.95 (18)	C15—C14—C6	132.0 (2)
N2—C3—C4	122.08 (18)	C15—C14—H14A	114.0
C2—C3—C4	116.97 (18)	C6—C14—H14A	114.0
C5—C4—C3	120.95 (18)	C14—C15—C19	117.09 (19)
C5—C4—H4A	119.5	C14—C15—C16	126.51 (19)
C3—C4—H4A	119.5	C19—C15—C16	116.33 (17)
C4—C5—C6	122.88 (18)	O2—C16—O3	123.6 (2)
C4—C5—H5A	118.6	O2—C16—C15	124.6 (2)
C6—C5—H5A	118.6	O3—C16—C15	111.83 (17)
C1—C6—C5	114.76 (17)	O3—C17—C18	107.4 (2)
C1—C6—C14	119.08 (18)	O3—C17—H17A	110.2
C5—C6—C14	126.15 (18)	C18—C17—H17A	110.2
N1—C7—O1	110.20 (18)	O3—C17—H17B	110.2
N1—C7—S1	126.16 (16)	C18—C17—H17B	110.2
O1—C7—S1	123.64 (15)	H17A—C17—H17B	108.5
N1—C8—H8A	109.5	C17—C18—H18A	109.5
N1—C8—H8B	109.5	C17—C18—H18B	109.5
H8A—C8—H8B	109.5	H18A—C18—H18B	109.5
N1—C8—H8C	109.5	C17—C18—H18C	109.5
H8A—C8—H8C	109.5	H18A—C18—H18C	109.5
H8B—C8—H8C	109.5	H18B—C18—H18C	109.5
N1—C9—H9A	109.5	O4—C19—O5	124.18 (19)
N1—C9—H9B	109.5	O4—C19—C15	125.08 (19)
H9A—C9—H9B	109.5	O5—C19—C15	110.74 (18)
N1—C9—H9C	109.5	O5—C20—C21	106.9 (2)
H9A—C9—H9C	109.5	O5—C20—H20A	110.3
H9B—C9—H9C	109.5	C21—C20—H20A	110.3
N2—C10—C11	113.09 (19)	O5—C20—H20B	110.3
N2—C10—H10A	109.0	C21—C20—H20B	110.3
C11—C10—H10A	109.0	H20A—C20—H20B	108.6
N2—C10—H10B	109.0	C20—C21—H21A	109.5
C11—C10—H10B	109.0	C20—C21—H21B	109.5
H10A—C10—H10B	107.8	H21A—C21—H21B	109.5
C10—C11—H11A	109.5	C20—C21—H21C	109.5
C10—C11—H11B	109.5	H21A—C21—H21C	109.5
H11A—C11—H11B	109.5	H21B—C21—H21C	109.5
			
C7—O1—C1—C2	−81.3 (2)	C1—O1—C7—S1	−0.5 (3)
C7—O1—C1—C6	102.6 (2)	C3—N2—C10—C11	82.5 (3)
O1—C1—C2—C3	−176.38 (17)	C12—N2—C10—C11	−87.6 (2)
C6—C1—C2—C3	−0.6 (3)	C3—N2—C12—C13	93.3 (3)
C12—N2—C3—C2	172.1 (2)	C10—N2—C12—C13	−96.7 (2)
C10—N2—C3—C2	2.7 (3)	C1—C6—C14—C15	−178.1 (2)
C12—N2—C3—C4	−7.9 (3)	C5—C6—C14—C15	2.9 (4)
C10—N2—C3—C4	−177.31 (19)	C6—C14—C15—C19	−178.4 (2)
C1—C2—C3—N2	−178.06 (19)	C6—C14—C15—C16	−1.6 (4)
C1—C2—C3—C4	2.0 (3)	C17—O3—C16—O2	0.4 (3)
N2—C3—C4—C5	177.70 (19)	C17—O3—C16—C15	179.54 (17)
C2—C3—C4—C5	−2.3 (3)	C14—C15—C16—O2	−97.5 (3)
C3—C4—C5—C6	1.3 (3)	C19—C15—C16—O2	79.4 (3)
C2—C1—C6—C5	−0.6 (3)	C14—C15—C16—O3	83.4 (3)
O1—C1—C6—C5	175.20 (17)	C19—C15—C16—O3	−99.7 (2)
C2—C1—C6—C14	−179.70 (19)	C16—O3—C17—C18	177.37 (19)
O1—C1—C6—C14	−3.9 (3)	C20—O5—C19—O4	2.0 (3)
C4—C5—C6—C1	0.2 (3)	C20—O5—C19—C15	−177.53 (17)
C4—C5—C6—C14	179.26 (19)	C14—C15—C19—O4	4.6 (3)
C9—N1—C7—O1	2.5 (3)	C16—C15—C19—O4	−172.5 (2)
C8—N1—C7—O1	−179.18 (18)	C14—C15—C19—O5	−175.81 (18)
C9—N1—C7—S1	−177.15 (17)	C16—C15—C19—O5	7.0 (2)
C8—N1—C7—S1	1.1 (3)	C19—O5—C20—C21	171.3 (2)
C1—O1—C7—N1	179.82 (16)		
